# Environmental Fate of 4-Methylbenzylidene Camphor: Adsorption Behavior on Textile-Derived Microplastic Fibers in Wastewater and Surface Water Systems

**DOI:** 10.3390/ma18163799

**Published:** 2025-08-13

**Authors:** Maja Vujić, Tijana Marjanović Srebro, Sanja Vasiljević, Tajana Simetić, Jelena Molnar Jazić, Jasmina Agbaba, Aleksandra Tubić

**Affiliations:** Department of Chemistry, Biochemistry and Environmental Protection, Faculty of Sciences, University of Novi Sad, Trg Dositeja Obradovića 3, 21000 Novi Sad, Serbia; maja.loncarski@dh.uns.ac.rs (M.V.); tijanam@dh.uns.ac.rs (T.M.S.); sanjav@dh.uns.ac.rs (S.V.); tajana.djurkic@dh.uns.ac.rs (T.S.); jelena.molnar@dh.uns.ac.rs (J.M.J.); aleksandra.tubic@dh.uns.ac.rs (A.T.)

**Keywords:** microplastic fibers, 4-methylbenzylidene camphor, chemisorption, adsorption, water matrix effects, environmental fate

## Abstract

This study investigates the adsorption behavior of 4-methylbenzylidene camphor (4-MBC), a persistent ultraviolet filter, onto microplastic fibers (MPFs) released from domestic textiles, under environmentally relevant conditions. Two types of MPFs were used: MPF A, a heterogeneous blend of synthetic and natural fibers, and MPF B, a uniform polyester source. Adsorption experiments were conducted in municipal wastewater, Danube River surface water, and laundry effluent. Kinetic data best fit the pseudo-second-order model (R^2^ > 0.95), and the Elovich model indicated chemisorption involving heterogeneous binding sites. MPF A exhibited superior adsorption capacities (qₑ = 85.4–90.1 µg/g) compared to MPF B (58.8–66.8 µg/g). Langmuir isotherms yielded maximum adsorption capacities of 204.9 µg/g for MPF A and 116.7 µg/g for MPF B (R^2^ = 0.929–0.977), while D–R isotherm energies (12.0–21.7 kJ/mol) confirmed specific interactions, such as π–π stacking and hydrogen bonding. Adsorption efficiency was highest in municipal wastewater (total organic carbon—TOC = 13.12 mg/L, electrical conductivity—EC = 1152 µS/cm), followed by laundry and surface waters. These findings emphasize the critical role of polymer composition and matrix complexity in pollutant transport, suggesting MPFs are effective transporters of hydrophobic micropollutants in aquatic systems.

## 1. Introduction

Microplastics (MPs), defined as plastic fragments smaller than 5 mm, are present in numerous environmental settings, including marine sediments, freshwater bodies, terrestrial regions, and agricultural soils [1,2,3,4]. Since their discovery by Thompson et al. (2004), MPs have been the subject of extensive scientific research due to their persistence and ecological consequences [5]. These particles have the ability to adsorb various chemical contaminants, both organic and inorganic. Organic pollutants commonly associated with MPs include polycyclic aromatic hydrocarbons (PAHs), polychlorinated biphenyls (PCBs), pesticides, and pharmaceutical compounds [6,7,8,9,10,11,12,13,14,15,16].

The concurrent presence of MPs and organic pollutants in diverse environmental compartments has become a significant issue, spurring research into MPs’ role as pollutant vectors [17,18]. Investigations suggest that MPs facilitate the transport of contaminants over large distances, potentially increasing their bioavailability and toxicological impact in aquatic ecosystems [19,20]. Moreover, MPs are often ingested by marine species, leading to the accumulation and transfer of harmful substances through the food chain [19,21,22,23].

A major contributor to microplastic pollution is the release of synthetic textile fibers, which originate primarily from laundering materials like polyester, nylon, and acrylic. During washing, these fibers enter wastewater systems and eventually infiltrate aquatic environments, significantly adding to microplastic pollution worldwide. The increasing global consumption of textiles is expected to drive a corresponding rise in the use of household washing machines, with over 840 million units already in operation worldwide [24,25]. The laundering of synthetic textiles is a major source of microfiber (MPF) emissions into wastewater; however, less than 30% of the global population has access to wastewater treatment plants (WWTPs), with significantly lower coverage in developing regions [26]. These regions also account for a disproportionately high share of synthetic textile consumption.

Despite wastewater treatment, WWTPs exhibit limited efficiency in retaining MPFs, particularly in conventional treatment systems [27,28,29]. Consequently, untreated and partially treated effluents contribute to the accumulation of MPFs in aquatic ecosystems, exacerbated by direct discharge through submarine outfalls [30,31,32]. Additionally, MPFs retained in WWTPs are often reintroduced into terrestrial environments via the application of sewage sludge as fertilizer [33].

Although studies have examined parameters influencing fiber release, including textile composition, detergent chemistry, and washing machine design, significant knowledge gaps persist [30,34,35,36]. Textile MPFs, being lightweight and highly mobile, have the potential to disperse extensively in water bodies, posing environmental and health hazards. The quantity of MPFs shed during washing depends on multiple variables, including fabric composition, garment wear, and washing parameters such as temperature, cycle duration, and detergent formulation [24,37,38,39].

The growing concern regarding emerging contaminants in aquatic environments has intensified research into anthropogenic pollutants and their interaction with various environmental matrices. Among these pollutants, 4-methylbenzylidene camphor (4-MBC), an organic ultraviolet (UV) filter widely used in sunscreen and personal care products, has been identified as a persistent contaminant with potential ecological and toxicological impacts. It primarily enters aquatic environments through untreated or inadequately treated wastewater and runoff from recreational areas [40,41,42]. To enhance UV-blocking efficacy, multiple UV filters are often combined in a single formulation, which contributes to elevated human and environmental exposure [40,43]. The global production and consumption of 4-MBC contribute to its continuous release into aquatic systems through industrial discharges, household wastewaters, and runoff, necessitating comprehensive studies to understand its environmental behavior and risks. Wastewater treatment plants (WWTPs), despite their advancements, are not entirely effective in removing 4-MBC due to its physicochemical properties, such as low water solubility and affinity for organic matter. Consequently, 4-MBC is often detected in surface waters, sediments, and even drinking water sources, with concentrations varying based on geographical location, wastewater treatment efficiency, and seasonal trends.

The presence of 4-MBC in surface waters, wastewater, and sediments has been extensively documented [44,45,46]. Reported concentrations of 4-MBC in surface waters range between 5 and 400 ng/L, with peak values observed in summer months. Contamination of groundwater occurs due to surface water infiltration and landfill leachate, with levels reaching up to 200 ng/L [45]. Additionally, wastewater treatment plants (WWTPs) have been identified as significant sources of 4-MBC discharge, with effluent concentrations reaching 2000 ng/L [44,45,46,47]. These contaminants exhibit low biodegradability and are inefficiently removed by conventional wastewater treatment processes [40,41,48,49]. Additionally, studies have shown that organic UV filters, including 2-hydroxy-4-methoxybenzophenone (BP-3), 4-methylbenzylidene camphor (4-MBC), and 2-Ethylhexyl-4-methoxycinnamate (EHMC), pose substantial health risks due to their endocrine-disrupting effects. Environmental risk assessments indicate that chronic exposure to these compounds adversely impacts aquatic habitats, particularly in regions with high recreational activity [50,51].

Due to its hydrophobic characteristics, 4-MBC has a tendency to sorb onto microplastic particles, accelerating its dispersion in aquatic environments. This interaction enhances the persistence of 4-MBC in the environment and raises concerns about its potential ecological consequences [7,52]. Furthermore, studies suggest that 4-MBC negatively affects marine species’ reproductive and developmental processes at environmentally relevant concentrations [53,54,55]. Its photostability, resulting from isomerization between (E)- and (Z)-isomers, further contributes to its environmental longevity [56,57].

Assessing the relationships between MPs with a focus on textile MPFs and 4-MBC in different water matrixes is essential for a comprehensive understanding of the mechanism of 4-MBC and microplastic pollution and its broader implications. Therefore, the main focus of this study was exploring the role of MPs as carriers for organic contaminants, with a particular focus on the adsorption and environmental fate of 4-MBC in wastewater collected from washing machines and surface water, the Danube River. Given its endocrine-disrupting potential and persistence across multiple ecosystems, assessing the interactions between MPs and UV filters is critical for determining their bioavailability, toxicological effects, and overall risks to environmental and human health.

## 2. Materials and Methods

### 2.1. Materials

Microplastic fibers (MPFs) from two differing sources were selected for the experimental work, reflecting real-world textile usage and fiber emission scenarios. The first category (MPF A) consisted of a varied composition of fibers shed from garments made of both synthetic (e.g., polyester, polyamide) and natural (cotton-based) textiles. Category two (MPF B) included only the consistent fibers released by a polyester blanket, selected because of its synthetic nature and high shedding. In both cases, fiber residues were obtained from the lint filter of a domestic tumble dryer following a standardized drying cycle, representing a common accumulation point for released microfibers in household settings.

For all experimental procedures, 4-methylbenzylidene camphor (4-MBC; C_18_H_22_O; CAS No. 36861-47-9) was used as the target analyte and was sourced as a certified reference material (TraceCERT^®^) from Sigma-Aldrich (St. Louis, MO, USA). Analytical-grade hexane and methanol (J.T. Baker, Avantor, Phillipsburg, NJ, USA) were utilized without further purification. Three water matrices were employed to reflect environmentally and domestically relevant conditions: (i) washing machine effluent collected immediately after a full laundry cycle, (ii) surface water sampled from a local freshwater system, and (iii) municipal wastewater. Detailed physicochemical parameters of selected water matrices are presented in Supplementary Table S1.

#### 2.1.1. Drying Garment Conditions

Microplastic fibers were generated under standardized and reproducible conditions designed to reflect typical household textile handling. Selected garments, including synthetic-cotton blends and a synthetic blanket, were subjected to a predefined laundering protocol using a domestic front-loading washing machine TESLA WF61032M (Tesla Electronics, Ljubljana, Slovenia). The washing cycle was conducted at 40 °C for 45 min under the “mixed fabrics” program, with a centrifugation speed of 800 rpm. Commercial detergent and fabric softener were applied in accordance with the manufacturer’s specifications. Four successive rinse cycles were included to emulate typical consumer behavior and minimize residual surfactant retention.

Immediately following laundering, the textiles were dried using a CANDY CSOE C8DF-S condenser-type tumble dryer (Candy Hoover Group, Brugherio, Italy). Drying was conducted for 60 min under the “low heat” setting, uniformly applied to all fabric types to ensure consistency across fiber sources. Upon completion of the drying cycle, lint fibers were carefully retrieved from the internal filter using stainless steel forceps previously cleaned with nitric acid and ultrapure water. The collected fibers were transferred into acid-washed, airtight borosilicate glass containers and stored under ambient laboratory conditions. All handling procedures were conducted in a contamination-controlled environment to minimize the risk of airborne microplastic exposure. Fibers were used in subsequent adsorption experiments without any chemical or physical modification.

#### 2.1.2. Microplastic Fiber Characterization

Prior to the adsorption experiments, microplastic fibers (MPFs) originating from synthetic blankets and a mixture of synthetic and natural cotton garments were thoroughly characterized to determine their morphological and chemical properties. Visual inspection was carried out using an optical microscope (Olympus BX41) (Evident Corporation (formerly Olympus Corporation), Hachioji, Tokyo, Japan) equipped with a Canon 1200D digital camera, operated at magnifications of 4× and 10×. Captured images were processed using ImageJ software, Version 1.53h, U.S. National Institutes of Health, Bethesda, MD, USA, to evaluate surface features, color, and morphology. Further morphological analysis was conducted using scanning electron microscopy (SEM; TM3030, Hitachi High-Technologies, Tokyo, Japan), which revealed that garment-derived fibers exhibited irregular, flattened, and heterogeneous structures, indicative of a mixed composition including natural fibers such as cotton or wool. In contrast, fibers collected from synthetic blankets displayed uniform, smooth, and cylindrical morphologies characteristic of synthetic polymers. Chemical identification of the MPFs was performed using Fourier-transform infrared spectroscopy (FTIR) in attenuated total reflectance (ATR) mode on a Nicolet iS20 spectrometer (Thermo Fisher Scientific, Waltham, MA, USA), with spectral data collected over a range of 4000–400 cm^−1^ and compared against reference spectra from the Hummel Polymer Library. The analysis confirmed the presence of cellulose-based materials in the garment fibers and polyester as the dominant polymer in the synthetic fibers. Thermogravimetric analysis (TGA) was conducted using a SDT Q600 analyzer (TA Instruments, New Castle, DE, USA) coupled with a Hiden Analytical HPR-20/QIC mass spectrometer (Hiden Analytical Ltd., Warrington, UK). Samples (1–2 mg) were heated from room temperature to 800 °C at a rate of 20 °C/min under a nitrogen atmosphere (flow rate: 50 cm^3^/min). The thermal degradation patterns corroborated the FTIR results, showing a two-step decomposition process for garment-derived fibers and a single, sharp degradation peak for blanket-derived fibers, consistent with pure polyester. These characterization results, which are further detailed in the Supplementary Materials (Figures S1–S4) and discussed in Vujić et al. (2024), provide essential insights into the structural and compositional heterogeneity of the MPFs and their expected influence on subsequent adsorption behavior [58].

#### 2.1.3. Contamination Prevention in Laboratory Settings

Due to the high risk of microplastic contamination in environmental sample analysis, strict contamination control measures were applied at every stage of the experimental process to ensure data integrity and reproducibility. All chemical reagents were pre-filtered using membrane filters (Carl Roth GmbH + Co. KG, Karlsruhe, Germany) with a pore size of 0.63 µm to eliminate the possibility of introducing microplastic particles from laboratory-grade chemicals. Only glass and metal apparatus were employed for sample preparation, filtration, and storage. Plastic materials were completely excluded to prevent inadvertent contamination.

All laboratory experiments were conducted inside a laminar flow hood, which was thoroughly cleaned before use with a combination of mechanical dust removal and disinfection using 70% ethanol. Laboratory personnel wore 100% cotton lab coats to minimize the release of synthetic fibers. To assess potential airborne contamination during processing, procedural blanks containing ultrapure water were handled in parallel with environmental samples under identical conditions.

Prior to each experiment, all laboratory materials, including beakers, forceps, filtration units, and Petri dishes, underwent an intensive cleaning protocol consisting of immersion in nitric acid (HNO_3_), followed by multiple rinses with distilled water. Cleaned items were wrapped in aluminum foil and stored in a contamination-free environment. Immediately before use, the same materials were re-rinsed with HNO_3_ and distilled water to eliminate any residual particulates and were subsequently covered with sterile paper towels until needed. Petri dishes used for storing filters were acid-cleaned, rinsed with ultrapure deionized water, dried in a dust-free environment, and kept sealed whenever possible. During weighing, filters were transferred into pre-weighed Petri dishes under the laminar flow hood and measured using a calibrated analytical microbalance (Mettler-Toledo International Inc., Columbus, OH, USA). All beakers containing solutions were kept covered with aluminum foil and uncovered only during essential steps to minimize exposure to airborne particles.

Two procedural blanks were subjected to the full treatment cycle, including all cleaning, filtering, handling, and weighing steps. In addition, an open blank filter was placed in a Petri dish within the laminar flow cabinet to continuously monitor the ambient contamination level during the entire experiment. Contamination detected in all blanks was below the method detection limit, confirming the effectiveness of the applied protocols.

### 2.2. Adsorption Experiments

#### 2.2.1. Adsorption Kinetics

The adsorption kinetics and mechanisms of 4-methylbenzylidene camphor (4-MBC) onto microplastic fibers (MPFs) sourced from two non-identical textiles were assessed via batch experiments conducted at 25 ± 1 °C under controlled laboratory conditions. A 4-MBC stock solution (10 mg in 10 mL methanol) was used to prepare the experimental suspensions: 50 mg of MPFs in 50 mL aqueous solution, yielding an initial 4-MBC concentration of 100 µg/L. The chosen concentration reflects environmentally relevant levels of 4-MBC previously reported in various aquatic compartments, particularly in treated and untreated municipal wastewater (typically ranging from 20–200 µg/L), as well as in urban-influenced surface waters (10–100 µg/L), depending on seasonal and regional variations. By selecting 100 µg/L, this study aimed to simulate a realistic concentration representative of both effluent-dominated water bodies and surface waters receiving diffuse pollution, thus enabling broader applicability of the findings across aquatic environments.

All suspensions were agitated using an orbital shaker (IKA^®^ KS 501 Digital) (IKA Werke GmbH & Co. KG, Staufen im Breisgau, Germany) at 150 rpm and maintained at 25 °C. All kinetic tests were performed in triplicate, with the pH of the solutions adjusted and held constant at 7.23 ± 0.06, corresponding to neutral conditions commonly encountered in freshwater and treated effluents. Aliquots were collected at specific time intervals (1, 2, 3, 4, 5, 6, 24, 48, 72, and 96 h). Microplastic fibers were subsequently separated from the aqueous phase by filtration through cellulose nitrate (CN) membrane filters with a pore size of 0.45 µm. The concentration of 4-MBC remaining in solution was determined by gas chromatography-mass spectrometry (GC–MS; Agilent 7890A/5975C) (Agilent Technologies, Inc., Santa Clara, CA, USA). The kinetic model parameters were determined by applying a nonlinear least-squares regression method to the experimental data, allowing for optimal fitting of the pseudo-first-order, pseudo-second-order, and Elovich models (Table S2).

#### 2.2.2. Adsorption Isotherms

The equilibrium adsorption characteristics of 4-MBC onto MPFs were systematically assessed through batch isotherm experiments conducted across a range of initial solute concentrations. Aqueous solutions containing 25, 50, 75, 100, 150, and 200 μg/L of 4-MBC were prepared, each with a constant MPF dose of 50 mg in 50 mL of solution. All experimental conditions, including pH and temperature (25 ± 1 °C), were consistent with those employed during the kinetic studies. Each suspension was agitated for 48 h to ensure that equilibrium was attained. Afterward, MPFs were removed by filtration through cellulose nitrate (CN) membrane filters with a pore size of 0.45 µm. The remaining 4-MBC concentration in the filtrate was determined using GC–MS. The adsorption behavior was interpreted using five classical isotherm models: Langmuir, Freundlich, Redlich–Peterson (R–P), Dubinin–Radushkevich (D–R), and Temkin. These models were employed to analyze surface heterogeneity, adsorption intensity, and monolayer capacity. Nonlinear least-squares regression was applied for model fitting, and the corresponding parameters are presented in Table S2.

#### 2.2.3. Analytical Conditions

Residual concentrations of 4-MBC were quantified following extraction by liquid–liquid partitioning with n-hexane. Analytical determinations were performed using a gas chromatography-mass spectrometry system (GC–MS; Agilent 7890A GC coupled with a 5975C MSD). Chromatographic separation was achieved using a DB-5MS capillary column (30 m × 0.25 mm × 0.25 µm). The oven temperature was initially set at 70 °C (held 0 min), ramped to 250 °C at 30 °C/min (held 5 min), and then further increased to 280 °C (held 0 min). The injector operated in splitless mode with a 2 µL injection volume. Electron impact ionization was applied at 70 eV, with the ion source and quadrupole maintained at 230 °C and 150 °C, respectively. The transfer line was held at 280 °C, with a solvent delay of 4.5 min. SIM and SCAN acquisition modes were used. The quantifier ion for 4-MBC was m/z 254, with confirmation ions at m/z 171 and 128. For EHMC, the quantifier and confirming ions were m/z 290, 178, and 161. Method performance showed recovery rates between 86% and 114%, with relative standard deviations below 15%. The method detection limit (MDL) was established at 50 ng/L.

In addition, the aqueous matrices were characterized in accordance with relevant ISO and SRPS standards. pH was measured using a WTW 340i pH meter equipped with a SenTix^®^21 electrode (SRPS H.Zi.111:1987) (Xylem Analytics Germany Sales GmbH & Co. KG (WTW), Weilheim, Germany). Dissolved organic carbon (DOC) was determined using a LiquiTOC II analyzer (Elementar, Langenselbold, Germany) per SRPS ISO 8245:2007. Total phosphorus and orthophosphate were analyzed by spectrophotometry using ammonium molybdate (SRPS EN ISO 6878:2008). Nitrite and nitrate concentrations were determined by molecular absorption spectrometry (SRPS EN 26777:2009) and sulfosalicylic acid spectrophotometry (SRPS ISO 7890-3:1994), respectively. Ammonia was measured using the Nesslerization method (SRPS ISO H.Zi.184:1974). Chemical oxygen demand (COD) was determined by the dichromate oxidation method (SRPS ISO 6060:1994).

#### 2.2.4. Data Analysis

All experimental data were processed, fitted, and graphically represented using OriginPro 8.5 (OriginLab Corporation, Northampton, MA, USA).

## 3. Results

### 3.1. Kinetic Modeling and Adsorption Behavior

**Figure 1:**
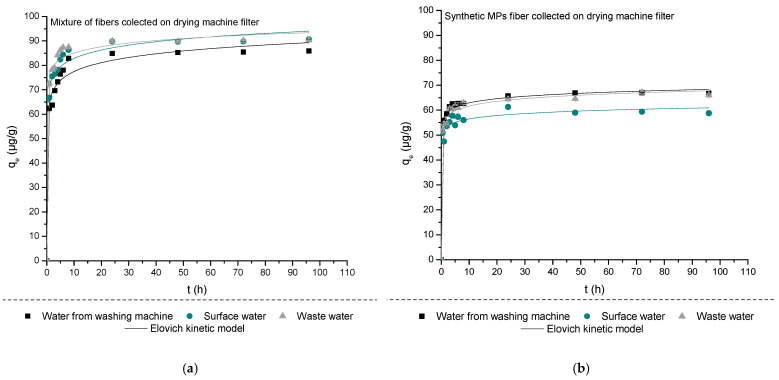
Non-linear regressions of kinetics data (initial concentration of 4-MBC: 100 µg/L, m_MPs fibers_: 50 mg, n = 3, mean value ± SD), obtained using Elovich model for 4-MBC on (**a**) MPF A and (**b**) MPF B.

The adsorption kinetics of 4-MBC on MPFs A and B in waters collected from washing machines, surface water, and municipal wastewater are presented in Figure 1 and Table S3. The adsorption of 4-methylbenzylidene camphor (4-MBC) onto both microplastic fibers (MPF A and MPF B) followed pseudo-second-order (PSO) kinetics across all tested water matrices, with R^2^ values exceeding 0.95. The theoretical equilibrium adsorption capacities (q_e_) derived from the PSO model closely matched the experimental values, confirming the model’s suitability [59,60,61]. In contrast, the pseudo-first-order (PFO) model systematically underestimated q_e_ values, indicating its limited applicability for systems governed by chemisorption. The adsorption efficiency of 4-MBC onto MPFs increased with contact time, showing rapid uptake within the first 6 h, followed by a slower approach to equilibrium. This behavior suggests a two-stage adsorption process involving fast surface adsorption and slower intraparticle diffusion. The adsorption kinetics were best described by the pseudo-second-order model, with high correlation coefficients (R^2^ > 0.97). The rate constants k_2_ for MPF A ranged from 0.044 to 0.071 g/µg·h, depending on the water matrix, while for MPF B, values ranged from 0.101 to 0.147 g/µg·h, indicating a higher adsorption rate for synthetic fibers. The corresponding equilibrium q_e_ ranged between 58.2 and 90.1 µg/g. These values and model fittings are summarized in Table S2 and confirm the chemisorption-driven mechanism of interaction.

The Elovich model also provided excellent fits (R^2^ = 0.968–0.996), especially in more chemically complex matrices, supporting the presence of heterogeneous binding sites with varying adsorption energies [62,63,64]. Compared to our previous findings in ultrapure water, the current results validate the robustness of the PSO model under environmentally relevant conditions and highlight the influence of matrix complexity on kinetic performance [58].

Chemisorption appears to be the primary mechanism for 4-MBC adsorption on MPF, as indicated by the strong Elovich fits and the PSO model’s superior performance, suggesting interactions with functional groups on the microplastic fiber surface. Considering 4-MBC’s physicochemical properties possible interactions that occur between 4-MBC and selected MPs fibers are: π–π stacking interactions, between the aromatic rings of 4-MBC and aromatic groups in the polymer backbone (e.g., in PET or PA microfibers); hydrophobic interactions, driven by the low water solubility of 4-MBC (log Kow ≈ 4.95), causing its partitioning onto nonpolar surfaces; hydrogen bonding or dipole–dipole interactions which may occur between the camphor carbonyl or methyl groups and oxygen-containing functional groups on MPFs (e.g., ester or amide moieties), and Van der Waals forces and physical trap of 4-MBC within surface roughness or fiber pores. These mechanisms are supported by previous studies on the adsorption of UV filters and pharmaceuticals onto polymers [43,52]. For instance, it is known that systems following PSO kinetics typically involve chemical bonding or surface-level interactions rather than simple diffusion or mass transfer. Moreover, a recent study by Pal et al. (2024) highlighted that sorption of aromatic pollutants is strongly enhanced on polymers containing π-electron-rich domains and hydrophobic surfaces [65].

In more complex matrices, such as municipal wastewater, the presence of natural organic matter, surfactants, and multivalent ions may further facilitate adsorption through bridging effects, surface charge modification, or competitive sorption pathways. Such matrix effects have been observed to enhance contaminant sorption in studies by Pal et al. (2024), Cui et al. (2023), and Torres-Agullo et al. (2021), suggesting that indirect interactions and conditioning layers may also play a role in real water systems [65,66,67].

MPF A consistently demonstrated higher adsorption capacities (q_e_ = 85.4–90.1 µg/g) compared to MPF B (q_e_ = 58.8–66.8 µg/g) across all matrices. This can be attributed to structural differences in the fibers, including higher specific surface area, more favorable surface polarity, and a greater density of reactive functional groups on MPF A. Elovich parameters (lower β values) further indicate that MPF A provides higher-energy adsorption sites, enabling more effective interactions with 4-MBC. These findings align with existing literature demonstrating that microplastic–organic pollutant interactions are highly dependent on polymer characteristics, particularly for aromatic and hydrophobic contaminants [65].

The composition of the water matrix strongly influenced both the kinetics and extent of 4-MBC adsorption. The highest adsorption efficiencies were observed in municipal wastewater, which exhibited elevated ionic strength (IS = 1152 µS/cm), total organic carbon (TOC = 13.12 mg/L), and dissolved metal concentrations. These conditions are known to enhance adsorption via electrostatic screening, increased pollutant solubility, and surface conditioning, which promote favorable interactions between the microplastic surface and hydrophobic molecules [65,68,69]. In contrast, surface water had lower conductivity (330 µS/cm) and TOC (2.86 mg/L), resulting in slightly lower kinetic rates and adsorption capacities. Despite the cleaner matrix, PSO and Elovich models still provided excellent fits, indicating that chemisorption dominates even in relatively pristine conditions. Adsorption results regarding the adsorption of 4-MBC on MPF A and MPF B in water obtained from a washing machine, although characterized by high TOC (31.22 mg/L), gave intermediate adsorption values. This may be attributed to the presence of surfactants, which can compete with 4-MBC for adsorption sites or alter fiber surface properties, potentially forming micelles that sequester 4-MBC and reduce its availability for direct interaction with MPs [70].

These findings confirm that microplastic fibers can act as effective carriers for hydrophobic organic contaminants such as 4-MBC, and that both polymer structure and water matrix composition play key roles in determining adsorption behavior. Compared to our previous study in ultrapure water (Vujić et al., 2024), the current results emphasize the need to account for real-world complexity when modeling contaminant fate [58]. The strong matrix-dependence observed in this study highlights the potential for enhanced pollutant transport in wastewater-impacted systems and underlines the importance of matrix-specific risk assessments for UV filters and other emerging contaminants.

### 3.2. Adsorption Isotherms and the Influence of Fiber Origin and Water Matrix Composition

**Figure 2:**
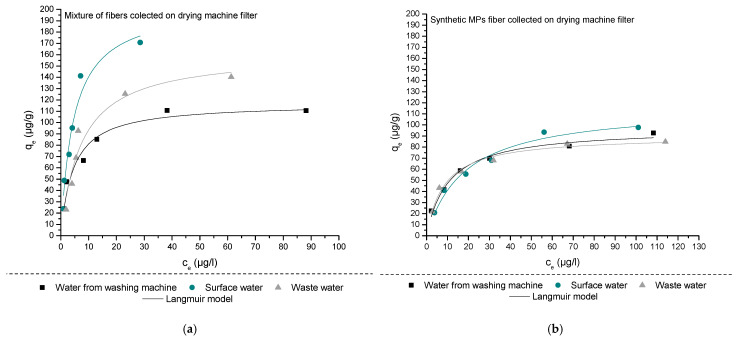
Adsorption isotherms plots (Initial concentrations of 4-MBC: 25, 50, 70, 100, 150, and 200 μg/L, m_MPs fibers_: 50 mg, n = 3, mean value ± SD) for 4-MBC on (**a**) MPF A and (**b**) MPF B.

The mechanism of adsorption of 4-methylbenzylidene camphor onto two selected types of microplastic fibers (MPFs), MPF A and MPF B, was assessed using five adsorption isotherm models: Langmuir, Freundlich, Redlich–Peterson (R–P), Dubinin–Radushkevich (D–R), and Temkin, and its results are presented in Figure 2 and Table S4. The Langmuir isotherm provided the best overall fit for the experimental data, with R^2^ values ranging from 0.929 to 0.977 across all water matrices and fiber types. The model’s applicability indicates that adsorption proceeds via monolayer coverage on relatively uniform binding sites. The calculated maximum adsorption capacities (q_max_) were higher for MPF A (116.8–204.9 µg/g) than for MPF B (73.9–116.7 µg/g), suggesting that the heterogeneous fiber blend in MPF A provides a greater diversity of surface functionalities, such as hydroxyl, amide, and ester groups that could enhance interaction with 4-MBC. These results align with previous findings (Vujić et al., 2024) that highlighted the role of mixed fiber compositions in providing more reactive or accessible sorption sites [58].

The Freundlich model yielded slightly lower correlation coefficients (R^2^ = 0.797–0.956) but supported the notion of heterogeneous surface adsorption, particularly in matrices with higher dissolved organic matter. Freundlich exponents (n_F_) below 1 across all systems suggested that adsorption was favorable but occurred on energetically diverse sites, which is consistent with the mixed polymer chemistry of MPF A and the complex nature of real water matrices [71,72,73]. The Redlich–Peterson model confirmed a predominantly Langmuir-like behavior with minor deviations, especially in laundry and municipal wastewater, where the presence of surfactants or natural organic matter may have altered surface energetics [71,74]. The D–R model further suggested that the adsorption mechanism was dominated by chemisorption, with calculated mean free energies (E_a_) ranging from 12.0 to 21.7 kJ/mol. This suggests the potential occurrence of stronger intermolecular interactions, such as hydrogen bonding, dipole–dipole forces, and π–π stacking, between the aromatic moiety of 4-MBC and the polymeric domains of MPF A, particularly those associated with polyamide and polyester fractions. Temkin isotherm results reinforced this interpretation, indicating moderate adsorption heats (56.5–145.0 J/mol) that reflect a gradual decrease in binding energy with increased surface coverage, characteristic of heterogeneously distributed binding sites [71,75,76]. The composition of the surrounding matrix significantly modulated adsorption behavior. Municipal wastewater, with high ionic strength (IS = 1152 µS/cm), TOC (13.12 mg/L), and dissolved multivalent ions, enhanced adsorption on both MPFs. These conditions facilitate pollutant–fiber interactions via electrostatic screening and surface conditioning effects. In contrast, surface water from the Danube River, with lower ionic strength (330 µS/cm) and TOC (2.86 mg/L), supported efficient but less extensive adsorption. Laundry wastewater, despite having the highest TOC (31.22 mg/L), resulted in intermediate adsorption, likely due to the presence of detergents and surfactants forming micelles that sequester 4-MBC, reducing its direct interaction with MPFs.

Overall, the results indicate that the adsorption of 4-MBC onto MPFs is governed by a combination of chemisorption and hydrophobic interactions, with polymer composition and matrix complexity both playing critical roles. The superior performance of MPF A highlights the advantage of fiber heterogeneity in enhancing pollutant binding through multiple sorption pathways. These findings contribute to a more nuanced understanding of how textile-derived MPFs interact with hydrophobic organic contaminants in diverse aquatic environments and emphasize the importance of considering real-world material sources and water chemistry when evaluating microplastic–pollutant interactions.

## 4. Conclusions

This study highlights the need to examine how microplastics and pollutants interact in realistic environmental settings, considering both plastic type and water complexity. Domestic microplastic fibers (MPFs) were shown to be effective sorbents for the ultraviolet filter 4-MBC, with adsorption governed primarily by chemisorption. In contrast to the uniform polyester MPF B, the heterogeneous composition of MPF A (synthetic and natural fibers) resulted in significantly improved adsorption, underscoring the impact of polymer diversity and surface characteristics. Equilibrium data were best described by Langmuir isotherms, indicating monolayer coverage. The D-R and Temkin models confirmed energetically heterogeneous sites and potential interactions like π–π stacking, hydrogen bonding, and hydrophobic partitioning. Water composition strongly impacted adsorption affinity with municipal wastewater (high in organic matter and multivalent ions), resulting increase in adsorption, while surface and laundry waters had distinct effects based on their ionic strength and surfactant content. Regarding the obtained results, future research should investigate 4-MBC desorption under dynamic conditions, the influence of microplastic weathering and biofilm formation, and co-contaminant effects. Furthermore, linking laboratory findings with in situ data and ecological risk assessments will be critical for understanding environmental fate and exposure pathways.

## Data Availability

The original contributions presented in this study are included in the article/supplementary material. Further inquiries can be directed to the corresponding author.

## Supplementary Materials

The following supporting information can be downloaded at: https://www.mdpi.com/article/10.3390/ma18163799/s1, Figure S1: Morphological characteristics obtained with an optical microscope of MPs fibers. (**a**) mixture of fibers collected on drying machine filter; (**b**) MPs fibers isolated from fibers collected on drying machine filter; Figure S2: SEM micrographs of MPs fibers (**a**) mixture of fibers collected on drying machine filter; (**b**) MPs fibers isolated from fibers collected on drying machine filter; Figure S3: FTIR spectra of MPs fiber A and MPs fiber B; Figure S4: Output of TGA analysis of MPs fibers (**a**) mixture of fibers collected on drying machine filter; (**b**) MPs fibers isolated from fibers collected on drying machine filter; Table S1: Characteristics of the water matrices; Table S2: Mathematical models used for modelling data obtained in kinetic and adsorption experiments; Table S3: Values calculated by kinetic models for adsorption of 4-MBC on MPs fibers in waters from washing machine, surface water and municipal wastewater; Table S4: Calculated parameters with different isotherm models for sorption of 4-MBC on MPs fibers in waters from washing machine, surface water and municipal wastewater. References [60,71,77,78,79] are cited in the Supplementary Materials.
