# Supplementary material for: Environmental Fate of 4-Methylbenzylidene Camphor: Adsorption Behavior on Textile-Derived Microplastic Fibers in Wastewater and Surface Water Systems
Source: Materials (Basel). 2025 Aug 13;18(16):3799. doi: 10.3390/ma18163799 (PMC12387943; doi:10.3390/ma18163799)
Supplement: Supplementary file 1 [file materials-18-03799-s001.zip › materials-3761104-supplementary.pdf]

materials

MDPI

Supplementary Materials

Environmental Fate of 4-Methylbenzylidene Camphor: Adsorption Behavior on Textile-Derived Microplastic Fibers in Wastewater and Surface Water Systems

Maja Vujić, Tijana Marjanović Srebro, Sanja Vasiljević, Tajana Simetić, Jelena Molnar Jazić, Jasmina Agbaba * and Aleksandra Tubić

Department of Chemistry, Biochemistry and Environmental Protection, Faculty of Sciences, University of Novi Sad, Trg Dositeja Obradovića 3, 21000 Novi Sad, Serbia
* Correspondence: jasmina.agbaba@dh.uns.ac.rs

Table S1. Characteristics of the water matrices.

Parameter	Surface Water	Municipal Wastewater	Laundry Wastewater
**pH**	7.9 ± 0.43	8.11 ± 0.05	8.10 ± 0.09
**Electrical conductivity 25 °C (μS/cm)**	330 ± 7	1152 ± 69	591 ± 22
**Turbidity (NTU)**	6.88 ± 2.94	5.75 ± 2.64	8 ± 0.09
**Total organic carbon (mg/L)**	2.86 ± 0.45	13.12 ± 3.68	31.22 ± 2.23
**HPK (mg C/L)**	9.6 ± 0.2	52 ± 10	132 ± 14
**Anionic surfactant (mg/L)**	< 0.03	9 ± 1.33	215 ± 3
**Fe (mg/L)**	0.024 ± 0.02	0.59 ± 0.35	< 0.14
**Mn(μg/L)**	16.8 ± 2.1	52.7 ± 3.31	< 2.34
**Ni (μg/L)**	3.71 ± 0.11	8.54 ± 0.36	< 2.15
**Zn (mg/L)**	0.009 ± 0.02	0.179 ± 0.03	< 0.023
**Cd (μg/L)**	< 0.15	< 0.15	< 0.15
**Cr (μg/L)**	1.73 ± 1.77	1.83 ± 0.31	< 0.90
**Cu (μg/L)**	7.02 ± 7.31	35.03 ± 17	< 0.90
**Pb (μg/L)**	< 5.9	< 5.9	< 5.9
**As (μg/L)**	2.31 ± 0.34	12 ± 0.82	< 2.6
**Hg (μg/L)**	< 0.5	< 0.5	< 0.5
**NH_3 (mg N/L)**	0.54 ± 0.2	4.35 ± 6.82	1.43 ± 0.86
**PO_4^{3-} (mg P/L)**	0.04 ± 0.03	1.19 ± 0.39	0.06 ± 0.03
**Total P (mg P/L)**	0.07 ± 0.06	1.38 ± 0.62	0.76 ± 0.03
**NO_2^- (mg N/L)**	< 0.002	0.24 ± 0.3	0.08 ± 0.05
**NO_3^- (mg N/L)**	2.001 ± 0.4	0.05 ± 0.01	0.04 ± 0.02

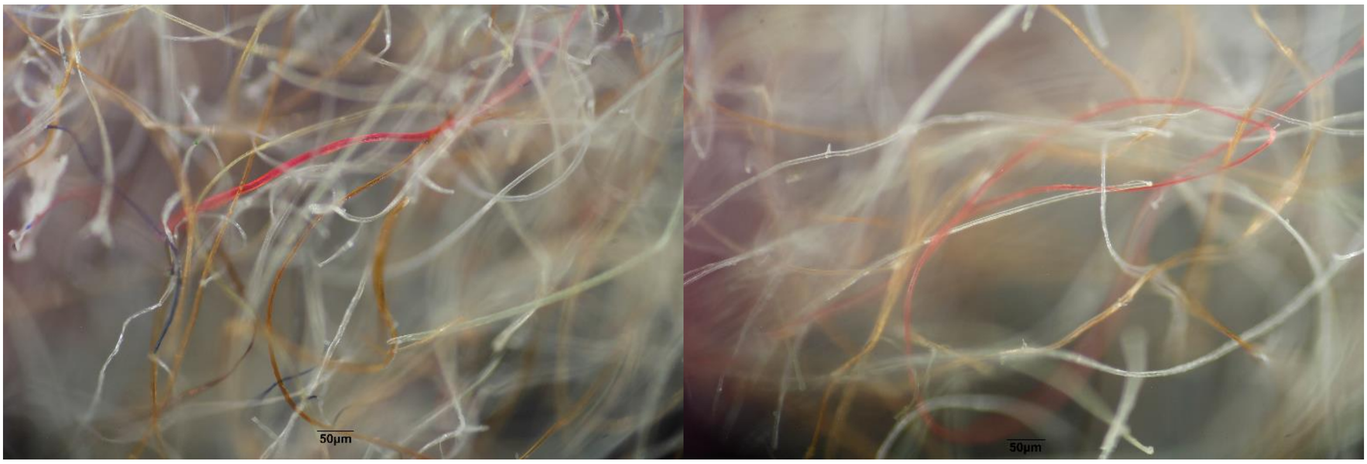

(**a**) (**b**)

Figure S1. Morphological characteristics obtained with an optical microscope of MPs fibers. (**a**) mixture of fibers collected on drying machine filter; (**b**) MPs fibers isolated from fibers collected on drying machine filter.

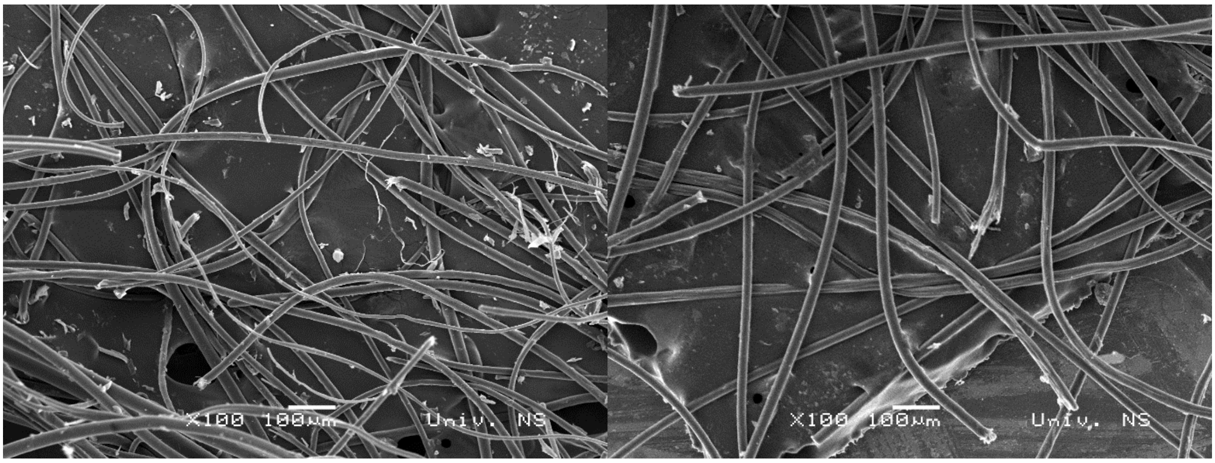

(a) **(b)**

Figure S2. SEM micrographs of MPs fibers (**a**) mixture of fibers collected on drying machine filter; (**b**) MPs fibers isolated from fibers collected on drying machine filter. Reprinted from Ref. [1].

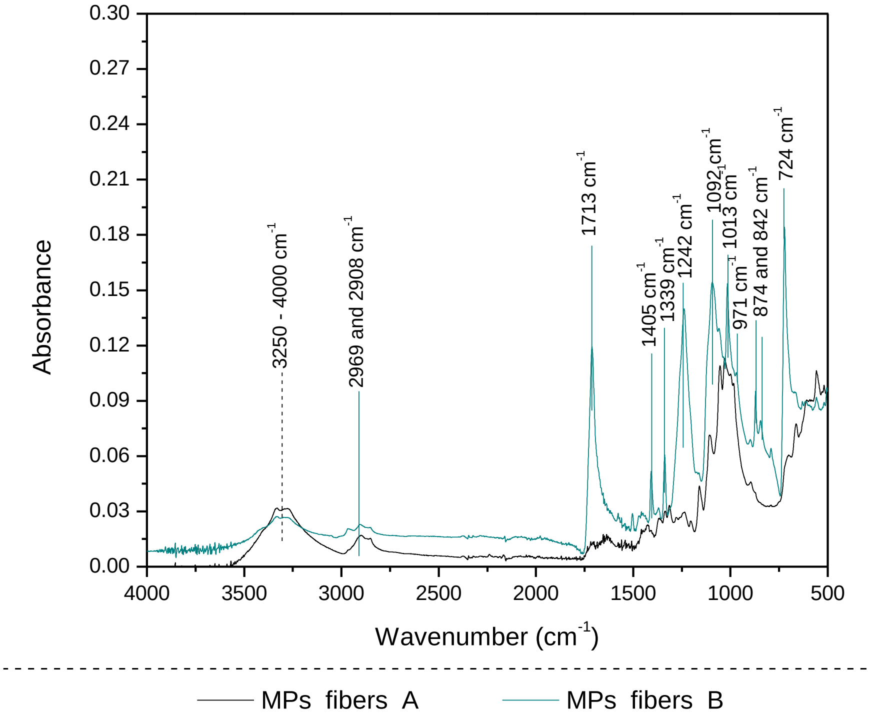

Figure S3. FTIR spectra of MPs fiber A and MPs fiber B

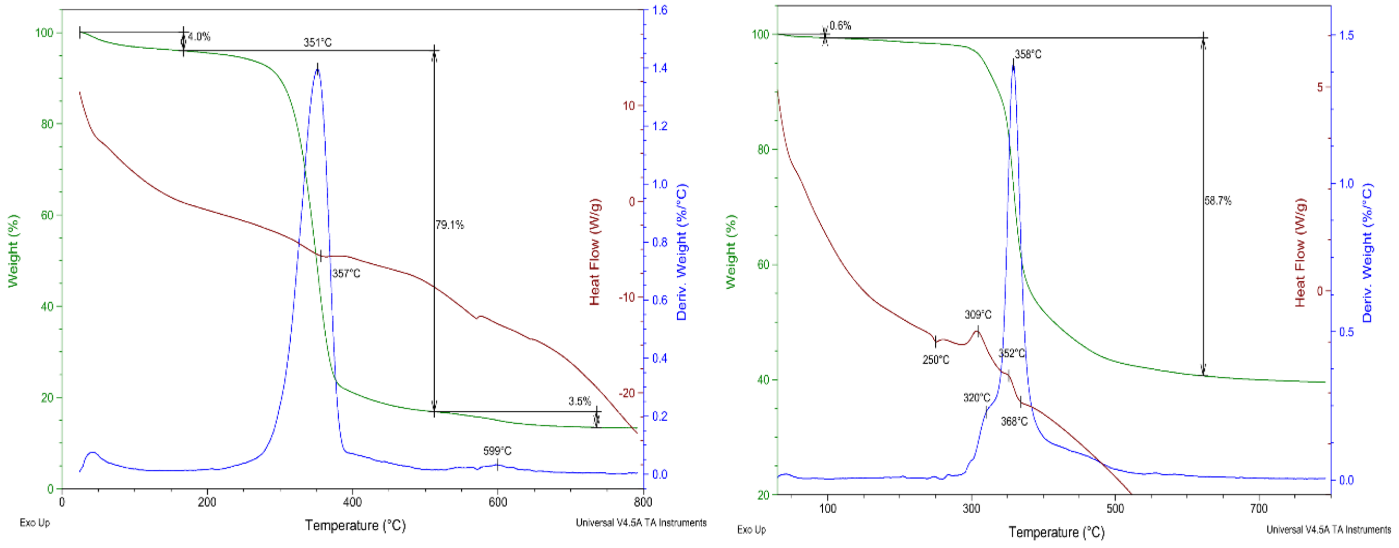

(a) **(b)**

Figure S4. Output of TGA analysis of MPs fibers (**a**) mixture of fibers collected on drying machine filter; (**b**) MPs fibers isolated from fibers collected on drying machine filter.

Table S2. Mathematical models used for modelling data obtained in kinetic and adsorption experiments.

Model	Equation	Linear Form	Reference
**Pseudo-first-order**	$\frac{dq_t}{dt} = k_1(q_e - q_t)$	$\log(q_e - q_t) = \log q_e - \frac{k_1}{2.303}t$	Wang and Guo (2020) [2]
**Pseudo-second-order**	$\frac{dq_t}{dt} = k_2(q_e - q_t)^2$	$\frac{t}{q_t} = \frac{1}{k_2 q_e^2} + \frac{1}{q_e}t$	
**Elovich**	$\frac{dq_t}{dt} = \alpha\, exp(-\beta q_t)$	$q_t = \frac{1}{\beta}\ln(\alpha\beta) + \frac{1}{\beta}\ln t$	Nanganoa et al. (2019) [3]
**Freundlich**	$q_e = K_F C_e^{n_F}$	$\log q_e = \log K_F + n_F \log C_e$	Worch, 2012 [4]
**Langmuir**	$q_e = \frac{K_L C_L}{1 + \alpha_L C_e}$	$\frac{C_e}{q_e} = \frac{\alpha_L}{K_L}C_e + \frac{1}{K_L}$	Foo and Hameed, 2010 [5]
**Temkin**	$q_e = \frac{RT}{b_T}\ln(A_T C_e)$	$q_e = \frac{RT}{b_T}\ln A_T + \frac{RT}{b_T}\ln C_e$	
**Dubinin-Redushkevich**	$q_e = q_s\, exp(-B\varepsilon^2)$	$\ln q_e = \ln q_s - B\varepsilon^2$	
**Redlich-Petersen**	$q_e = \frac{K_R C_R}{1 + \alpha_R C_e^{\beta}}$	$\ln\left[K_R \frac{C_e}{q_e} - 1\right] = \ln \alpha_R + \beta \ln C_e$	

Table S3. Values calculated by kinetic models for adsorption of 4-MBC on MPs fibers in waters from washing machine, surface water and municipal wastewater.

		**Pseudo-First Order**			**Pseudo-Second Order**					**Elovich**		
**MPs Fibers**	**Water Matrixes**	**k_1 (h^{-1})**	**q_e (µg/g)**	**R^2**	**k_2 (g/µg h)**	**h (µg/g h)**	**q_e (theoretical) (µg/g)**	**q_e (experimental) (µg/g)**	**R^2**	**α µg/g h**	**β µg/g**	**R^2**
**MPF A**	Water from washing machine	2.46	78.2	0.887	0.044	299.5	82.5	85.4	0.952	1.41×10^6	0.19	0.968
	Surface water	2.46	83.8	0.931	0.048	368.3	87.6	89.7	0.978	1.33×10^7	0.21	0.983
	Municipal wastewater	3.15	85.7	0.954	0.071	557.4	88.6	90.1	0.985	4.39×10^9	0.27	0.986
**MPF B**	Water from washing machine	3.65	63.2	0.967	0.125	526.5	64.9	66.8	0.988	7.38×10^{10}	0.42	0.996
	Surface water	3.86	56.6	0.954	0.147	497.9	58.2	58.8	0.977	6.08×10^{11}	0.51	0.982
	Municipal wastewater	3.23	62.0	0.957	0.101	415	64.1	66.0	0.984	2.02×10^9	0.37	0.991

k_1—rate constant of first-order sorption; k_2 ($g{\cdot}mg^{-1}{\cdot}min^{-1}$): Pseudo-second-order rate constant; q_e—adsorption capacity; R^2—correlation coefficient; k_2—rate constant of second-order sorption; α ($mg{\cdot}g^{-1}{\cdot}min^{-1}$): Initial adsorption rate (Elovich model); β ($g{\cdot}mg^{-1}$): Desorption constant (Elovich model).

Table S4. Calculated parameters with different isotherm models for sorption of 4-MBC on MPs fibers in waters from washing machine, surface water and municipal wastewater.

MPs Fibers	Water Matrixes	Freundclih Model			Langmuir Model			Redlich-Petersen Model			Dubinine-Radushkevich Model			Temkin Model		
		K_F (μg/g)/(μg/L)^n	n_F	R^2	K_L (L/μg)	q_{max} (μg/g)	R^2	K_R (L/g)	β	R^2	B	E_a	R^2	b_T (J/mol)	A_T (L/g)	R^2
MPF A	Water from washing machine	38.3	0.26	0.860	0.23	116.8	0.951	33.0	0.94	0.942	0.0011	21.7	0.918	119.9	3.65	0.942
	Surface water	50.7	0.38	0.831	0.22	204.9	0.955	29.7	1.45	0.972	0.0011	20.9	0.961	56.5	2.15	0.939
	Municipal wastewater	36.9	0.34	0.797	0.14	161.5	0.929	17.4	1.20	0.927	0.0018	16.6	0.961	71.6	1.28	0.905
MPF B	Water from washing machine	22.6	0.31	0.956	0.09	97.03	0.977	15.5	0.85	0.990	0.0023	14.7	0.902	136.3	1.41	0.990
	Surface water	17.5	0.39	0.936	0.05	116.7	0.974	7.62	0.91	0.969	0.0035	12.0	0.919	101.8	0.61	0.975
	Municipal wastewater	24.2	0.28	0.906	0.12	90.16	0.977	13.1	0.94	0.976	0.0020	15.9	0.957	145.0	1.61	0.972

q_m—Maximum adsorption capacity (Langmuir model); K_L—Langmuir equilibrium constant; K_F - Freundlich capacity constant; n—Freundlich intensity constant; A_T—Temkin equilibrium binding constant; b_T—Temkin heat of adsorption; K_R, β—Redlich–Peterson model constants; B—Dubinin–Radushkevich constant; Ea—Mean adsorption energy (D-R model).

References

1. Maja, V.; Sanja, V.; Teresa, R.-S.; Jasmina, A.; Zoran, Č.; Jelena, R.; Aleksandra, T. Improving of an easy, effective and low-cost method for isolation of microplastic fibers collected in drying machines filters. *Sci. Total. Environ.* **2023**, *892*, 164549, https://doi.org/10.1016/j.scitotenv.2023.164549.
2. Wang, J.; Guo, X. Adsorption kinetic models: Physical meanings, applications, and solving methods. *J. Hazard. Mater.* **2020**, *390*, 122156, https://doi.org/10.1016/j.jhazmat.2020.122156.
3. Nanganoa, L.T.; Merlain, G.T.; Ndi, J.N.; Ketcha, J.M. Removal of ammonium ions from aqueous solution using hydroxy-sodalite zeolite. *Asian J. Green Chem.* **2019**, *3*, 169–186, https://doi.org/10.22034/ajgc.2018.140227.1083.
4. Worch, E. *Adsorption Technology in Water Treatment*; Walter de Gruyter GmbH: Berlin, Germany, 2012, https://doi.org/10.1515/9783110240238.
5. Foo, K.; Hameed, B. Insights into the modeling of adsorption isotherm systems. *Chem. Eng. J.* **2010**, *156*, 2–10, https://doi.org/10.1016/j.cej.2009.09.013.
